# Supplementary material for: Bidirectional alterations in brain temperature profoundly modulate spatiotemporal neurovascular responses in-vivo
Source: Commun Biol. 2023 Feb 17;6:185. doi: 10.1038/s42003-023-04542-6 (PMC9935519; doi:10.1038/s42003-023-04542-6)
Supplement: Supplementary file 2 — Supplementary Information [file 42003_2023_4542_MOESM2_ESM.pdf]

Supplementary Table 1: 2s Baseline and response values

	44°C	40 °C	37 °C	Ambient	20 °C	10 °C	6 °C	Single Factor Anova Result
Actual brain temperature (°C)	39.8 ± 0.2	37.5 ± 0.2	36.0 ± 0.2	28.4 ± 0.8	23.4 ± 0.3	15.3 ± 0.5	12.5 ± 0.6	F=551.1 df=6 $p=6.4x10^{-27}$
Change in brain temperature (oC)	-0.019 ± 0.004	-0.008 ± 0.003	0.008 ± 0.002	0.051 ± 0.007	0.096 ± 0.020	0.054 ± 0.019	0.013 ± 0.004	F=9.93 df=6 $p=2.9x10^{-6}$
Brain tissue O_2 measurement (mmHg)	37.6 ± 3.0	33.6 ± 4.0	30.6 ± 3.9	12.4 ± 3.1	15.1 ± 2.4	9.1 ± 1.2	8.2 ± 1.0	F=13.69 df=6 $p=4.3x10^{-7}$
Change in Tissue oxygen (Fractional)	1.021 ± 0.007	1.036 ± 0.008	1.052 ± 0.024	1.046 ± 0.017	1.029 ± 0.013	0.977 ± 0.010	0.991 ± 0.004	F=3.51 df=6 p=0.009
Hbt Onset (s)	0.54 ± 0.14	0.64 ± 0.07	0.45 ± 0.05	0.79 ± 0.13	1.81 ± 0.13	3.40 ± 0.74	4.82 ± 1.77	F=4.15 df=6 p=0.003
Hbt Time to peak (s)	2.59 ± 0.05	2.79 ± 0.22	2.92 ± 0.19	3.83 ± 0.49	5.52 ± 0.27	9.96 ± 1.84	13.46 ± 6.76	F=11.52 df=6 $p=6.3x10^{-7}$
Hbt Peak magnitude (Fractional)	1.028 ± 0.006	1.040 ± 0.006	1.042 ± 0.008	1.051 ± 0.013	1.056 ± 0.011	1.01 ± 0.002	1.003 ± 0.001	F=5.48 df=6 p=0.0005
Hbr dip magnitude (Fractional)	1.009 ± 0.002	1.008 ± 0.002	1.005 ± 0.001	1.007 ± 0.001	1.014 ± 0.001	1.019 ± 0.003	1.011 ± 0.001	F=7.01 df=6 $p=7.2x10^{-5}$
LFP Magnitude (Volts)	0.47 ± 0.04	0.52 ± 0.03	0.56 ± 0.01	0.54 ± 0.03	0.69 ± 0.04	0.64 ± 0.06	0.48 ± 0.05	F=3.29 df=6 p=0.015
LFP Minima (Volts)	-0.0017 ± 0.0003	-0.0022 ± 0.0002	-0.0025 ± 0.0003	-0.0020 ± 0.0002	-0.0019 ± 0.0002	-0.0011 ± 0.0002	-0.0006 ± 0.0001	F=5.65 df=6 p=0.00066
MUA Magnitude (Spikes total during impulse)	156.46 ± 5.35	187.77 ± 5.5	187.74 ± 5.63	230.72 ± 7.11	243.52 ± 10.24	164.31 ± 16.58	61.62 ± 8.94	F=33.38 df=6 $p=1.17x10^{-12}$

Supplementary Table 2: 16 Baseline and response values

	44 °C	40 °C	37 °C	Ambient	20 °C	10 °C	6 °C	Single Factor Anova Results
Actual brain temperature (°C)	39.4 ± 0.3	37.5 ± 0.2	36.0 ± 0.2	28.9 ± 0.7	23.4 ± 0.2	15.1 ± 0.4	12.5 ± 0.5	F=652.7 df=6 $p=2.1x10^{-34}$
Change in brain temperature (oC)	-0.01 ± 0.003	-0.003 ± 0.002	0.015 ± 0.006	0.146 ± 0.016	0.252 ± 0.022	0.086 ± 0.021	0.076 ± 0.018	F=33.45 df=6 $p=4.25x10^{-13}$
Brain tissue O_2 measurement (mmHg)	38.3 ± 4.1	35.3 ± 3.9	30.9 ± 3.6	14.0 ± 3.0	13.2 ± 2.1	8.2 ± 1.2	8.1 ± 1.1	F=16.447 df=6 $p=6.7x10^{-9}$
Change in Tissue oxygen (Fractional)	1.068 ± 0.025	1.119 ± 0.038	1.1153 ± 0.053	1.175 ± 0.093	1.081 ± 0.038	0.980 ± 0.033	0.967 ± 0.014	F=2.31 df=6 p=0.054
Hbt Onset (s)	0.47 ± 0.22	0.47 ± 0.09	0.56 ± 0.05	0.87 ± 0.20	1.86 ± 0.14	4.66 ± 0.31	6.51 ± 0.41	F=89.24 df=6 $p=9.8x10^{-20}$
Hbt Time to peak (s)	3.07 ± 0.14	3.27 ± 0.12	3.77 ± 0.15	9.71 ± 1.89	10.13 ± 1.40	17.48 ± 1.36	23.79 ± 3.42	F=19.21 df=6 $p=9.0x10^{-10}$
Hbt Peak magnitude (Fractional)	1.031 ± 0.005	1.046 ± 0.007	1.050 ± 0.009	1.079 ± 0.013	1.096 ± 0.014	1.020 ± 0.002	1.003 ± 0.001	F=8.959 df=6 $p=6x10^{-6}$
Hbr dip magnitude (Fractional)	1.011 ± 0.003	1.004 ± 0.001	1.008 ± 0.001	1.009 ± 0.001	1.013 ± 0.002	1.024 ± 0.002	1.022 ± 0.002	F=11.63 df=6 $p=4.0x10^{-7}$
LFP Magnitude (Volts)	0.27 ± 0.03	0.31 ± 0.03	0.34 ± 0.03	0.42 ± 0.02	0.47 ± 0.04	0.16 ± 0.03	0.087 ± 0.01	F=21.01 df=6 $p=2.77x10^{-10}$
LFP Minima (Volts)	-0.0014 ± 0.0002	-0.0019 ± 0.0003	-0.0020 ± 0.0003	-0.0020 ± 0.0002	-0.0016 ± 0.0002	-0.0004 ± 0.0001	-0.0002 ± 0.00005	F=13.02 df=6 $p=1.09x10^{-7}$
MUA Magnitude (Spikes total during impulse)	152.80 ± 14.58	169.90 ± 6.97	173.93 ± 8.98	213.54 ± 8.58	216.59 ± 6.65	76.85 ± 15.93	31.09 ± 7.50	F=43.81 df=6 $p=7.58x10^{-15}$

Supplementary Table 3: Significant post-hoc Tukey test results

	Two Seconds (DF all 33)	Sixteen seconds(DF all 35)
Actual brain temperature	44°C-37°C,p=0.0004<br>44°C-Amb, p<0.0001<br>44°C-20 °C, p<0.0001<br>44°C-10 °C, p<0.0001<br>44°C-6 °C, p<0.0001<br>40°C-Amb, p<0.0001<br>40°C-20 °C, p<0.0001<br>40°C-10 °C, p<0.0001<br>40°C-6 °C, p<0.0001<br>37°C-Amb, p<0.0001<br>37°C-20°C, p<0.0001<br>37°C-10°C, p<0.0001<br>37°C-6 °C, p<0.0001<br>Amb -20 °C, p<0.0001<br>Amb -10 °C, p<0.0001<br>Amb -6°C, p<0.0001<br>20°C-10 °C, p<0.0001<br>20°C-6°C, p<0.0001<br>10°C-6 °C, p=0.0033	44°C-40°C,p=0.0474<br>44°C-37°C, p<0.0001<br>44°C-Amb, p<0.0001<br>44°C-20 °C, p<0.0001<br>44°C-10 °C, p<0.0001<br>44°C-6 °C, p<0.0001<br>40°C-Amb, p<0.0001<br>40°C-20 °C, p<0.0001<br>40°C-10 °C, p<0.0001<br>40°C-6 °C, p<0.0001<br>37°C-Amb, p<0.0001<br>37°C-20°C, p<0.0001<br>37°C-10°C, p<0.0001<br>37°C-6 °C, p<0.0001<br>Amb -20 °C, p<0.0001<br>Amb -10 °C, p<0.0001<br>Amb -6°C, p<0.0001<br>20°C-10 °C, p<0.0001<br>20°C-6°C, p<0.0001<br>10°C-6 °C, p=0.0023
Change in brain temperature	44°C-Amb, p=0.0177<br>44°C-20 °C, p<0.0001<br>44°C-10 °C, p=0.0123<br>40°C-Amb, p=0.0316<br>40°C-20 °C, p<0.0001<br>40°C-10 °C, p=0.0212<br>37°C-20°C, p=0.0003<br>20°C-6°C, p=0.0008	44°C-Amb, p<0.0001<br>44°C-20 °C, p<0.0001<br>44°C-10 °C, p=0.0035<br>44°C-6 °C, p=0.011<br>40°C-Amb, p<0.0001<br>40°C-20 °C, p<0.0001<br>40°C-10 °C, p=0.0075<br>40°C-6 °C, p=0.0208<br>37°C-Amb, p<0.0001<br>37°C-20°C, p<0.0001<br>Amb -20 °C, p=0.0009<br>20°C-10 °C, p<0.0001<br>20°C-6°C, p<0.0001
Actual Tissue oxygen	44°C-Amb, p=0.0002<br>44°C-20 °C, p=0.0011<br>44°C-10 °C, p<0.0001<br>44°C-6 °C, p<0.0001<br>40°C-Amb, p=0.0006<br>40°C-20 °C, p=0.0032<br>40°C-10 °C, p<0.0001<br>40°C-6 °C, p<0.0001<br>37°C-Amb, p=0.0039<br>37°C-20°C, p=0.019<br>37°C-10°C, p=0.0005<br>37°C-6 °C, p=0.0002	44°C-Amb, p=0.0001<br>44°C-20 °C, p<0.0001<br>44°C-10 °C, p<0.0001<br>44°C-6 °C, p<0.0001<br>40°C-Amb, p=0.0009<br>40°C-20 °C, p=0.0005<br>40°C-10 °C, p<0.0001<br>40°C-6 °C, p<0.0001<br>37°C-Amb, p=0.0125<br>37°C-20°C, p=0.0078<br>37°C-10°C, p=0.0004<br>37°C-6 °C, p=0.0003

Change in Tissue oxygen	37°C-10 °C, p=0.017<br>Amb -10 °C, p=0.037	NO Sig post-hoc results
Hbt onset	44 °C-6 °C, p=0.0352<br>40 °C-6 °C, p=0.0167<br>37 °C-6 °C, p=0.019<br>Amb-6°C, p=0.0229	44°C-20 °C, p=0.0078<br>44°C-10 °C, p<0.0001<br>44°C-6 °C, p<0.0001<br>40°C-20 °C, p=0.0077<br>40°C-10 °C, p<0.0001<br>40°C-6 °C, p<0.0001<br>37°C-20 °C, p=0.0149<br>37°C-10 °C, p<0.0001<br>37°C-6 °C, p<0.0001<br>Amb -10 °C, p<0.0001<br>Amb -6°C, p<0.0001<br>20°C-10 °C, p<0.0001<br>20°C-6°C, p<0.0001<br>10°C-6 °C, p=0.0002
Hbt time to Peak	44°C-10 °C, p=0.0095<br>44 °C-6 °C, p<0.0001<br>40°C-10 °C, p=0.0039<br>40 °C-6 °C, p<0.0001<br>37 °C-10 °C, p=0.0047<br>37 °C-6 °C, p<0.0001<br>Amb -10 °C, p=0.019<br>Amb -6°C, p<0.0001<br>20 °C-6 °C, p=0.011	44°C-10 °C, p<0.0001<br>44 °C-6 °C, p<0.0001<br>40 °C-10 °C, p<0.0001<br>40 °C- 6 °C, p<0.0001<br>37 °C-10 °C, p=0.0001<br>37 °C-6 °C, p<0.0001<br>Amb -6°C, p<0.0001<br>20 °C-6 °C, p=0.0001
Hbt magnitude	Amb -6°C, p=0.031<br>20 °C-10 °C, p=0.0122<br>20 °C-6 °C, p=0.0024	44 °C -Amb, p=0.0076<br>44°C-20 °C, p= 0.0001<br>40 °C-Amb, p=0.0273<br>40°C-20 °C, p<0.0001<br>37°C-20 °C, p=0.0002<br>Amb -10°C, p=0.0256<br>Amb -6°C, p=0.0005<br>20 °C-10 °C,p<0.0001<br>20 °C-6 °C, p<0.0001
Hbr Dip Magnitude	44°C-10 °C, p=0.0181<br>40°C-10 °C, p=0.0017<br>37°C-20 °C, p=0.0196<br>37°C-10 °C, p<0.000<br>Amb -10°C, p=0.0008	44°C-10 °C, p=0.0015<br>44 °C-6 °C, p=0.01<br>40°C-10 °C, p<0.0001<br>40 °C -6 °C, p<0.0001<br>37°C-10 °C, p=0.0002<br>37°C -6 °C, p=0.0013<br>Amb -10°C, p=0.0005<br>Amb -6°C, p=0.0032<br>20 °C -10 °C,p=0.0123
LFP Magnitude	44°C-20 °C, p=0.0393<br>20 °C-6 °C, p=0.0213	44 °C–Amb, p=0.0146<br>44°C-20 °C, p=0.0007<br>44°C-6 °C, p=0.0017<br>40°C-20 °C, p=0.0078<br>40°C-10 °C, p=0174<br>40°C-6 °C, p=0.0001<br>37 °C -20 °C, p=0.0418

		37 °C -10 °C, p=0.0029<br>37 °C -6°C, p<0.0001<br>Amb -10 °C, p<0.0001<br>Amb -6°C, p<0.0001<br>20 °C -10 °C, p<0.0001<br>20 °C -6 °C, p<0.0001
LFP Minima	40°C-10 °C, p=0.0468<br>40 °C - 6 °C, p=0.0012<br>37 °C -10 °C, p=0.0055<br>37 °C -6°C, p=0.0001<br>Amb -6°C, p=0.0037<br>20 °C -6 °C, p=0.01	44°C-10 °C, p=0.03<br>44°C-6 °C, p=0.0042<br>40°C-10 °C, p=0.0004<br>40°C-6 °C, p<0.0001<br>37°C-10 °C, p=0.0001<br>37°C-6 °C, p<0.0001;<br>Amb -10 °C, p<0.0001<br>Amb -6°C, p<0.0001<br>20 °C -10 °C, p=0.0036<br>20 °C -6 °C, p=0.0004
MUA Magnitude	44 °C -Amb, p=0.0012<br>44°C-20 °C, p= 0.0001<br>44°C-6°C, p<0.0001<br>40°C-20 °C, p=0.009<br>40°C-6°C, p=<0.0001<br>37°C-20 °C, p=0.009<br>37°C-6°C, p=<0.0001<br>Amb -10 °C, p=0.0012<br>Amb -6°C, p<0.0001<br>20°C-10 °C, p<0.0001<br>20°C-6°C, p<0.0001<br>10°C-6°C, p<0.0001	44 °C -Amb, p=0.004<br>44°C-20 °C, p= 0.0023<br>44°C-10°C, p=0.0002<br>44°C-6°C, p<0.0001<br>40°C-20 °C, p= 0.0475<br>40°C-10°C, p<0.0001<br>40°C-6°C, p<0.0001<br>37°C-10°C, p<0.0001<br>37°C-6°C, p<0.0001<br>Amb-10°C, p<0.0001<br>Amb-6°C, p<0.0001<br>20°C-10°C, p<0.0001<br>20°C-6°C, p<0.0001
