# Supplementary material for: Bidirectional alterations in brain temperature profoundly modulate spatiotemporal neurovascular responses in-vivo
Source: Commun Biol. 2023 Feb 17;6:185. doi: 10.1038/s42003-023-04542-6 (PMC9935519; doi:10.1038/s42003-023-04542-6)
Supplement: Supplementary file 3 — Reporting Summary [file 42003_2023_4542_MOESM3_ESM.pdf]

nature portfolio

Corresponding author(s): Jason Berwick
Last updated by author(s): Nov 2, 2022

Reporting Summary

Nature Portfolio wishes to improve the reproducibility of the work that we publish. This form provides structure for consistency and transparency in reporting. For further information on Nature Portfolio policies, see our Editorial Policies and the Editorial Policy Checklist.

Statistics

For all statistical analyses, confirm that the following items are present in the figure legend, table legend, main text, or Methods section.

n/a	Confirmed	
☐	☒	The exact sample size (n) for each experimental group/condition, given as a discrete number and unit of measurement
☐	☒	A statement on whether measurements were taken from distinct samples or whether the same sample was measured repeatedly
☐	☒	The statistical test(s) used AND whether they are one- or two-sided<br>*Only common tests should be described solely by name; describe more complex techniques in the Methods section.*
☐	☒	A description of all covariates tested
☐	☒	A description of any assumptions or corrections, such as tests of normality and adjustment for multiple comparisons
☐	☒	A full description of the statistical parameters including central tendency (e.g. means) or other basic estimates (e.g. regression coefficient) AND variation (e.g. standard deviation) or associated estimates of uncertainty (e.g. confidence intervals)
☐	☒	For null hypothesis testing, the test statistic (e.g. F, t, r) with confidence intervals, effect sizes, degrees of freedom and P value noted<br>*Give P values as exact values whenever suitable.*
☒	☐	For Bayesian analysis, information on the choice of priors and Markov chain Monte Carlo settings
☒	☐	For hierarchical and complex designs, identification of the appropriate level for tests and full reporting of outcomes
☒	☐	Estimates of effect sizes (e.g. Cohen's d, Pearson's r), indicating how they were calculated

Our web collection on statistics for biologists contains articles on many of the points above.

Software and code

Policy information about availability of computer code

Data collection	2D-OIS data was collected using a bespoke lab built system, Neural activity was collected using a Tucker-Davis Technology System, Oxygen and Temperature data was collected using the Oxylite Pro System.
Data analysis	All analysis was performed using lab developed Matlab code which is available on request.

For manuscripts utilizing custom algorithms or software that are central to the research but not yet described in published literature, software must be made available to editors and reviewers. We strongly encourage code deposition in a community repository (e.g. GitHub). See the Nature Portfolio guidelines for submitting code & software for further information.

Data

Policy information about availability of data

All manuscripts must include a data availability statement. This statement should provide the following information, where applicable:
- Accession codes, unique identifiers, or web links for publicly available datasets
- A description of any restrictions on data availability
- For clinical datasets or third party data, please ensure that the statement adheres to our policy

Datasets used in the current study are available in the DRYAD repository, https://doi.org/10.5061/dryad.n2z34tmzq

Human research participants

Policy information about studies involving human research participants and Sex and Gender in Research.

Reporting on sex and gender	NA
Population characteristics	NA
Recruitment	NA
Ethics oversight	NA

Note that full information on the approval of the study protocol must also be provided in the manuscript.

Field-specific reporting

Please select the one below that is the best fit for your research. If you are not sure, read the appropriate sections before making your selection.

☒ Life sciences ☐ Behavioural & social sciences ☐ Ecological, evolutionary & environmental sciences

For a reference copy of the document with all sections, see nature.com/documents/nr-reporting-summary-flat.pdf

Life sciences study design

All studies must disclose on these points even when the disclosure is negative.

Sample size	No Power calculation was performed for this dataset as we did not know the range of differences we would see from the change in brain temperature. Group sizes were based upon previous experimental work with acute experimental studies in the group and around the field.
Data exclusions	All animals were used in the study.
Replication	Results were consistently replicated across all animals and sessions and this is reflected in the levels of significance.
Randomization	Statement included : Randomization and blinding were not performed in this study
Blinding	Statement included : Randomization and blinding were not performed in this study

Reporting for specific materials, systems and methods

We require information from authors about some types of materials, experimental systems and methods used in many studies. Here, indicate whether each material, system or method listed is relevant to your study. If you are not sure if a list item applies to your research, read the appropriate section before selecting a response.

Materials & experimental systems

n/a	Involved in the study	
☒	☐	Antibodies
☒	☐	Eukaryotic cell lines
☒	☐	Palaeontology and archaeology
☐	☒	Animals and other organisms
☒	☐	Clinical data
☒	☐	Dual use research of concern

Methods

n/a	Involved in the study	
☒	☐	ChIP-seq
☒	☐	Flow cytometry
☒	☐	MRI-based neuroimaging

Animals and other research organisms

Policy information about studies involving animals; ARRIVE guidelines recommended for reporting animal research, and Sex and Gender in Research

Laboratory animals	Included in the text : Six female Hooded Lister rats weighing between 230 to 330g were kept in a temperature-controlled environment (20-22C) under a 12-hour dark/light cycle.

Wild animals	None
Reporting on sex	We have reported that we have used only one sex.
Field-collected samples	NA
Ethics oversight	Surgical and experimental procedures were regulated by the UK Home Office, in accordance with the Animal (Scientific procedures) Act 1986.

Note that full information on the approval of the study protocol must also be provided in the manuscript.
